# Effects of NH_4_^+^/citrate complexing agent ratio on Ni–Mo and Ni–Mo–O electrodeposits from ammonium citrate baths

**DOI:** 10.3389/fchem.2022.942423

**Published:** 2022-08-30

**Authors:** Dung T. To, Sun Hwa Park, Min Joong Kim, Hyun-Seok Cho, Nosang V. Myung

**Affiliations:** ^1^ Department of Chemical and Biomolecular Engineering, University of Notre Dame, Notre Dame, Korea; ^2^ Smart Devices Team, Korea Research Institute of Standards and Science, Deajeon, Korea; ^3^ Hydrogen Research Department, Korea Institute of Energy Research, Daejeon, Korea

**Keywords:** Ni–Mo alloys, Ni–Mo–O composites, induced co-deposition, Hull cell, electrodeposition

## Abstract

To understand the effect of complexing agents (i.e., ammonium and citrate) in nickel–molybdenum electrodeposition, calculation of the concentration of various Ni and Mo species as a function of pH and initial concentration of metal ions and complexing agents was performed. In addition, linear sweep voltammetry and Hull cell experiments were systematically investigated to understand the effect of current density and ammonium-to-citrate ratio to film compositions, morphology, and crystallinity. The results indicated that Ni(NH_3_)_3_^2+^ played a critical role in induced co-deposition mechanism of Ni–Mo alloys, which involved the reduced Ni and absorbed H atoms. Microstructure analysis of deposits indicated that the transition from smooth laminarly grown amorphous Ni–Mo–O composites to columnar and nanocrystalline metallic Ni–Mo alloys with a globular structure as the ammonium-to-citrate molar ratio increases. The highest Mo content of alloys was as high as 19 at%, and up to 70 at% O was present in the composites.

## Introduction

Nickel is an element in the first row of transition metals, which has been alloying with other transition metals at different stoichiometries for various applications. Among them, Ni–Mo alloys are well-known for the excellent corrosion resistance, which makes them a potential alternative for the electroplated chromium (Brooman, 2004; Mousavi et al., 2016). With a good mechanical strength, Ni–Mo alloys have also been used as the substrate for different purposes. For example, Hastelloy, a commercialized Ni–Mo alloy, is the substrate for synthesis of superconductor by ion beam-assisted deposit (Yamada et al., 2009). In addition, Ni–Mo alloys show their superior hydrogen evolution reaction (HER) activities and stability in comparison to other Ni-based alloys (e.g. Ni–Mo > Co–W > Co–Mo > Ni–W > Ni–Co > Ni–Fe > Ni–Cr) (Tasic et al., 2011; Safizadeh et al., 2015). Each application requires distinct material properties of Ni–Mo alloys (e.g., composition, morphology, and crystallinity). Bai et al. reported the phase diagram of Ni–Mo constructed from multilayered Ni–Mo films using ion mixing technique (Bai and Liu, 1993). The phases of Ni–Mo alloys vary from amorphous to mixed phase and crystalline phase, depending on the Mo content and post-thermal treatment conditions. Also, transition of the crystalline structure of alloys from face-centered cubic to tetragonal, orthorhombic, and body-centered cubic takes place as Mo content increases (Bai and Liu, 1993). Therefore, tunability of the material properties of Ni–Mo alloys becomes important to facilitate them for applications.

Ni–Mo alloys have been synthesized by various methods, such as electrodeposition (Ernst et al., 1955; Higashi et al., 1976; Fukushima and Higashi, 1978; Nee et al., 1988; BeBtowska-Lehman, 1990; Arul Raj, 1992; Podlaha et al., 1993; Fan et al., 1994; Podlaha and Landolt, 1996; Chassaing et al., 2004; Allahyarzadeh et al., 2016), arc melting (Rengakuji et al., 1994), e-beam evaporation (Zhang and Liu, 1994), and sputtering (Kawashima et al., 1997). However, the number and the range of studied parameters are relatively small because the investigation requires high cost and time consumption, especially under harsh synthesis conditions. Hull cell, an electrodeposition technique, becomes a promising means for the high throughput investigation of synthesis parameters. Similar to other efficient electrodeposition techniques, Hull cell not only provides the tunability of morphology and composition but also requires a simple setup and near ambient operating conditions. Owing to the diagonal configuration between the cathode and anode, Hull cell allows the deposition with a wide range of continuous current densities by only a single experiment.

Ni–Mo alloys are electrodeposited *via* induced co-deposition, which was first introduced by Brenner (1963). The reduction of Mo is induced by the reduction of Ni, with the assistance of complexing agent because Mo reduction occurs at much more negative potential than HER as shown in equations (Brooman, 2004; Yamada et al., 2009; Mousavi et al., 2016).

$$\begin{array}{cc} H^{+}+2e^{-}\to H_{2} & E^{o}=0V\;\mathrm{vs}.\;\mathrm{RHE} \end{array},$$ (1)

$$\begin{array}{cc} Ni^{2+}+2e^{-}\to Ni & E^{o}=-0.257V\;\mathrm{vs}.\;\mathrm{RHE} \end{array},$$ (2)

$$\begin{array}{cc} MoO_{4}^{2-}+4H_{2}O+6e^{-}\to Mo+8OH^{-} & E^{o}=-0.913\;V\;\mathrm{vs}.\;\mathrm{RHE} \end{array}.$$ (3)

Many polycarboxylic acids and their derivatives have been used as the complexing agent for Ni–Mo co-deposition, such as citrate (Ernst et al., 1955; Nee et al., 1988; Chassaing et al., 1989a; BeBtowska-Lehman, 1990; Arul Raj, 1992; Podlaha et al., 1993; Fan et al., 1994; Podlaha and Landolt, 1996; Chassaing et al., 2004; Allahyarzadeh et al., 2016) and tartrate (Higashi et al., 1976; Fukushima and Higashi, 1978). Among them, citrate is the most common complexing agent, which tunes the reduction rate of Ni^2+^ ions and correspondingly controls the composition of Ni–Mo alloys. Moreover, the polymerization of molybdate ions to form isopolymolybdate species is suppressed in the presence of citrate ions, favoring the reduction of Mo (Shinoda et al., 1997; Murase et al., 2000). As Mo possesses multiple valence states, Mo easily becomes partially reduced, consequently forming Ni–Mo–O deposits. In addition, ammonium hydroxide is commonly used in both citrate and tartrate bath for Ni–Mo co-deposition, but its role remains unclear (Ernst et al., 1955; Higashi et al., 1976; Fukushima et al., 1979; Nee et al., 1988; BeBtowska-Lehman, 1990; Arul Raj, 1992; Podlaha et al., 1993; Fan et al., 1994; Podlaha and Landolt, 1996; Chassaing et al., 2004; Allahyarzadeh et al., 2016). Since the complexing agents are essential for the co-deposition of Ni–Mo alloys, the overall electrodeposition consists of two key processes: the complexation and the reduction reactions. The complexation between metal precursors and complexing agents generates many different species. Only chemically active species participate in the reduction reactions at the interface of electrolyte and electrode. Thereby, it is necessary to study the distribution of species after complexation to maximize the fraction of chemically active species for Ni–Mo co-deposition. Owing to the complicated two-step process, a combination of computation and experiment is a good approach, which was demonstrated by researchers from various fields (Sharma et al., 2019; Zhu et al., 2019).

Effects of various synthesis parameters on the formation of Ni–Mo alloys and Ni–Mo–O composites were investigated using Hull cell and linear sweep voltammetry in a series of work. In the first part, the fraction of complex species at equilibrium is simulated using commercially available software (i.e., MATLAB) to understand the complexation of metals and complexing agents and predict the solution composition to maximize the chemically active species for reduction reactions. Also, the effect of complexing agents (i.e., citrate and ammonium) on the film composition, morphology, and crystal structure was experimentally studied and correlated with simulated data to elucidate the deposition mechanism.

## Experimental methods

### Electrodeposition

The electrolyte was prepared by dissolving the metal precursors and the complexing agents in the sequence of sodium citrate dihydrate (ACS reagent, Sigma Aldrich), nickel sulfate hexahydrate (Certified ACS, Fisher Scientific), sodium molybdate dihydrate (ACS reagent, Sigma Aldrich), and ammonium hydroxide (ACS reagent, Sigma Aldrich). The solution pH was corrected using NaOH and H_2_SO_4_. Circular gold-coated Cu electrode with an opening area of 0.65 cm^2^ and the commercially brass plate (267 ml brass cathode, Kocour) were used as the working electrode and substrate for the linear sweep voltammetry (LSV) and Hull cell electrodeposition. The LSV was conducted using the three-electrode system (i.e., a saturated Ag/AgCl (4M KCl) and a Pt-coated titanium plate as the reference and counter electrodes, respectively) with a Princeton Applications VMP2 potentio/gavalnostat. Potential was swept from open circuit potential to −2 V, with respect to the reference electrode at the scan rate of 5 mV/s. The electrodeposition employed Pt-coated Ti mesh as anode in the 267 ml Hull cell and powered by Hewlett Packard 6655 A DC power supply. Before the reaction, the substrate was cleaned with 1 M H2SO4, rinsed with deionized water, and blow dried with nitrogen gas.

### Material characterization

The morphology of the Ni–Mo and Ni–Mo–O thin films were observed by using a scanning electron microscope (Thermo Fisher Scientific Prisma E SEM). Energy dispersive X-ray spectroscopy (EDS) was used to characterize the composition of the thin films. The oxidation states of molybdenum in the composites were examined by X-ray photoelectron spectroscopy (XPS) using PHI VersaProbe II. The crystal structure of the structures was examined by powder X-ray diffraction (XRD, Bruker D8 Advance), with copper (*λ* = 1.5405 Å) as anticathode and 0.02-degree increments from 30 to 80°. The Psd Voigt fitting algorithm from Origin Pro software was utilized for the peak fitting. The grain size was calculated using the Scherrer equation, where D_hlk_ is the crystalline size, the constant K is 0.9, L is the ratio of the area under the peak and the peak height, and *θ* is the Bragg angle.

$$D_{hlk}=\frac{K\lambda }{Lcos\theta }$$

## Simulating the fraction of metal complex species

### Complexation of nickel and molybdenum ions with citrate and ammonium

As a tribasic organic acid, citric acid can disassociate into three hydronium ions and associated anions at different deprotonation constants. Depending on pH, the fraction of disassociated species and thereby fraction of complex species forming upon the addition of metal cations (e.g., nickel) will vary. Although ammonium ion only disassociates once to form an ammonia molecule and a hydronium ion, ammonia can complex with nickel ions at different stoichiometries. The possible dissociation and complexation between nickel and two complexing agents are compiled into Table 1, with their corresponding equilibrium constants.

**TABLE 1:** Equilibrium constant of deprotonation of complexing agents (citric acid and ammonium ions) and complexation of nickel ions with complexing agents (Hedwig et al., 1980; Dean and Lange, 1999; Open Textbook, 2011).

Deprotonation of citric acid	K_a_
H_3_Cit ↔ H_2_Cit^−^ + H^+^	10^–2.94^
H_2_Cit- ↔ HCit^2-^ + H^+^	10^–4.37^
HCit^2-^ ↔ Cit^3-^ + H^+^	10^–5.72^
Cit^3-^ ↔ H_-1_Cit^4-^ + H^+^	10^–11.8^
Deprotonation of ammonium ions	
NH_4_^+^→ NH_3_ + H^+^	10^–9.25^
Complexation of Ni (II) and citrate ions	
Ni^2+^ + H_2_Cit^−^ → NiH_2_Cit^+^	10^1.8^
Ni^2+^ + HCit^2-^ → NiHCit	10^3.6^
Ni^2+^ + Cit^3-^ → NiCit^-^	10^5.6^
NiCit^−^ + Cit^3-^ → NiCit_2_^4-^	10^2.4^
Complexation of Ni (II) and ammonia	
Ni^2+^ + NH_3_ → [Ni(NH_3_)]^2+^	10^2.80^
Ni^2+^ + 2NH_3_ → [Ni(NH_3_)_2_]^2+^	10^5.04^
Ni^2+^ + 3NH_3_ → [Ni(NH_3_)_3_]^2+^	10^6.77^
Ni^2+^ + 4NH_3_ → [Ni(NH_3_)_4_]^2+^	10^7.96^
Ni^2+^ + 5NH_3_ → [Ni(NH_3_)_5_]^2+^	10^8.71^
Ni^2+^ + 6NH_3_ → [Ni(NH_3_)_6_]^2+^	10^8.74^

When molybdenum element is introduced, the complexity of the system becomes escalated because of not only the second metal cation but also the polymerization of molybdate ions and the complexation with both nickel and citrate as shown in Table 2. The interaction between molybdenum and citrate can possibly form 17 complexes with different stoichiometries as shown in Supplementary Table S1. With the same fashion as citrate species, the presence and distribution of molybdenum species are pH-dependent.

**TABLE 2:** Equilibrium constant of polymerization of molybdate ions, complexation of citrate and Mo (IV) ion, and reaction of Ni and Mo (IV) ions (Murase et al., 2000; Murase et al., 2004).

Polymerization of molybdate ions	K_a_
7MoO_4_^2-^ + 8H^+^ → Mo_7_O_24_^6-^ + 4H_2_O	10^53.76^
7MoO_4_^2-^ + 9H^+^ → HMo_7_O_24_^5-^ + 4H_2_O	10^59.67^
7MoO_4_^2-^ + 10H^+^ → H_2_Mo_7_O_24_^4-^ + 4H_2_O	10^63.72^
Complexation of citrate and Mo (VI) ions	
4MoO_4_^2-^ + 11H^+^ + 4Cit^3-^ ↔ (MoO_4_)_4_H_11_Cit_4_^9-^	10^77.45^
Formation of Ni(II)-Mo(VI) heteropolymolybdate ion	
Ni^2+^ + 6MoO_4_^2-^ + 6H^+^ → NiMo_6_O_24_H_6_^4-^	10^44.35^

### Algorithm of the MATLAB program

The complexing reactions in Tables 1, 2 indicate that the fraction of species in the system consisting of citrate, ammonium, nickel, and molybdenum is determined not simply by the pH but also with the initial concentrations of nickel, molybdenum, and citrate precursors. Due to the multiple complex interactions among the components, a MATLAB program was developed to simulate the fraction of species corresponding to a set of initial concentrations and pH. By employing the simulation data, the correlation of the composition of electrolyte before and after complexation was determined to narrow down the initial electrolyte conditions for promoting the presence of chemically active complexes. The algorithm of the program that is based on the material balance with respect to nickel, molybdenum, ammonium, and citrate ions is explained in more details in the Supplemental Information. In addition, the concentration of H_-1_Cit^4-^ was presumably negligible due to a much smaller deprotonation constant compared to the first three constants of citric acid. Since the concentration of H^+^ and Cit^3-^ ions are inversely proportional, the concentrations of Mo (IV)-citrate complexes are negligible even with high complexing constants. Therefore, only (MoO_4_)_4_H_11_Cit_4_^9-^ was considered as Mo (IV)-citrate complex in the material balance.

**FIGURE 1:**
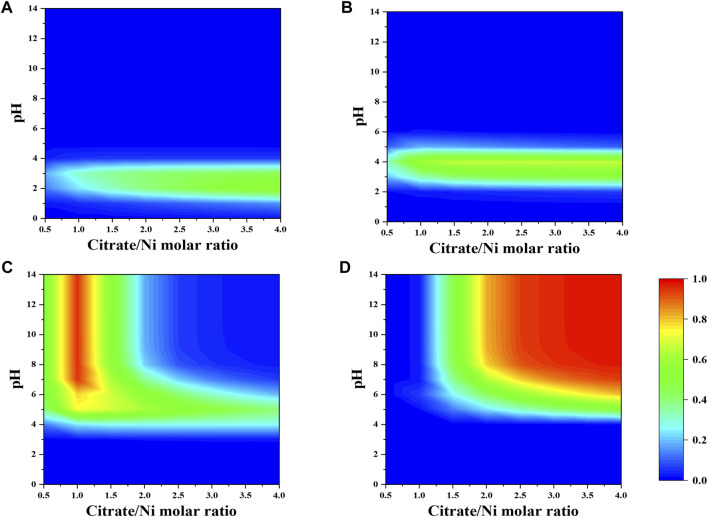
Fraction of nickel-citrate complexes **(A)** NiH_2_Cit^+^, **(B)** NiHCit, **(C)** NiCit^−^, and **(D)** NiCit_2_^4-^ as a function of citrate-to-nickel molar ratio and pH in the absence of NH_4_^+^.

**FIGURE 2:**
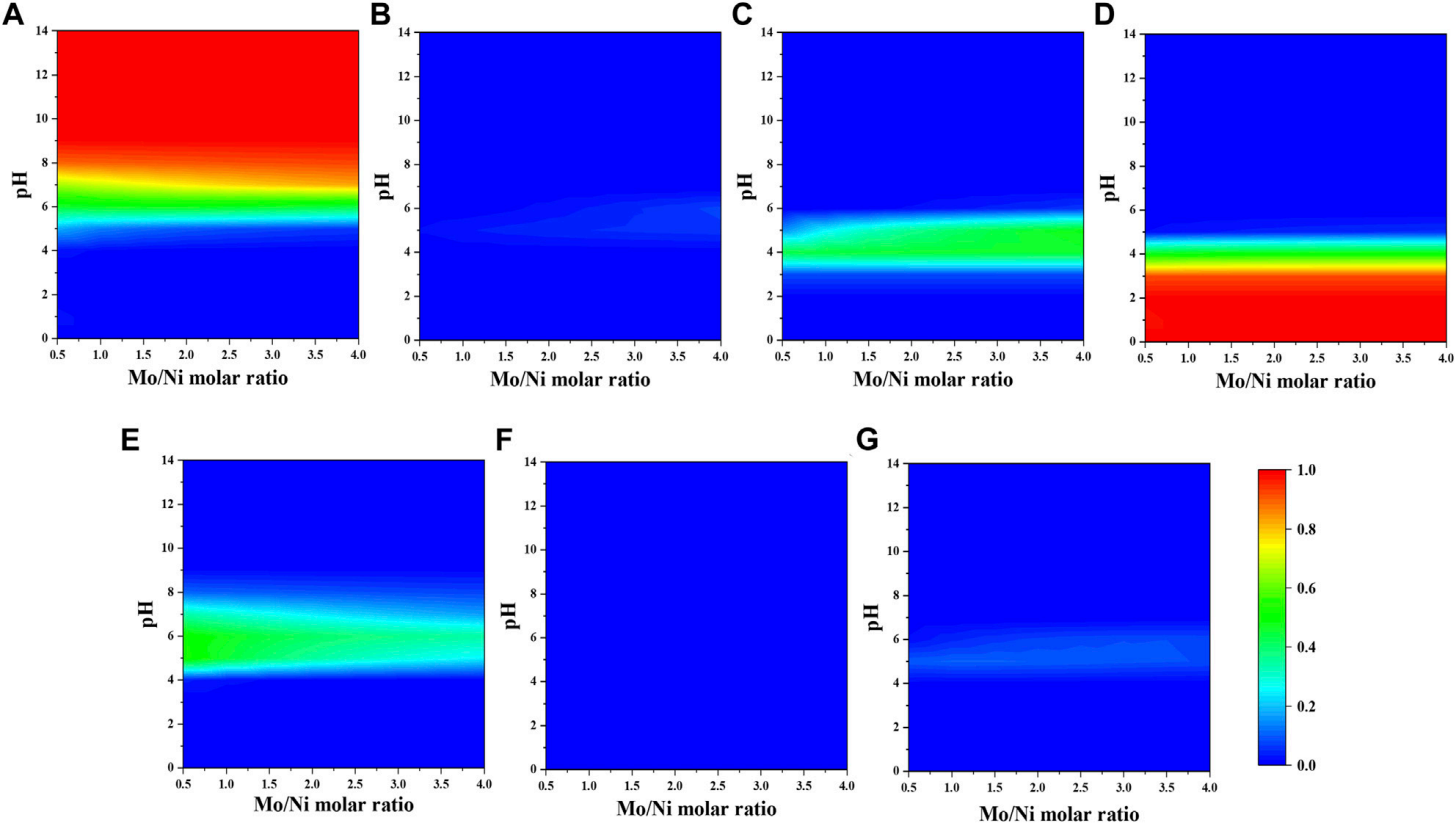
Fraction of molybdenum-citrate and nickel–molybdenum complexes **(A)** MoO_4_^2-^, **(B)** M_7_O_24_^6-^, **(C)** HMO_7_O_24_^5-^, **(D)** H_2_Mo_7_O_24_^4-^, **(E)** MoO_4_HCit^4-^, **(F)** (MoO_4_)_4_H_11_Cit_4_^9-^, and **(G)** NiMo_6_O_24_H_6_^4-^ as a function of citrate-to-nickel molar ratio and pH in the absence of NH_4_^+^.

Initial concentrations of precursors in which nickel ion was fixed at 0.1 M while Mo-to-Ni and citrate-to-Ni molar ratios were varied from 0.5 to 4.0 were used as the input of the simulation program. The contour plots are used to correlate the simulated fraction of species with pH, and the molar ratio of precursors are as shown in Figures 1, 2.

As shown in Figure 1, the dominant nickel–citrate complexes vary with pH and citrate-to-nickel molar ratio. The fraction of NiH_2_Cit^+^ and NiHCit species is barely dependent on the precursor molar ratio and mostly present in the acidic pH between two and five. In contrast, the majority of NiCit^−^ and NiCit_2_^4-^ species occurs in the alkaline pH and at the precursor molar ratio below 1.5 and above 2.0, respectively.

The fraction of molybdenum species mainly depends on pH (Figure 2). Two species with the considerable fractions are MoO_4_^2-^ (Figure 2A) and H_2_Mo_7_O_24_^4-^ (Figure 2D) at alkaline and acidic pH, respectively. The neutral pH solution contains molybdenum species with similar fractions.

**FIGURE 3:**
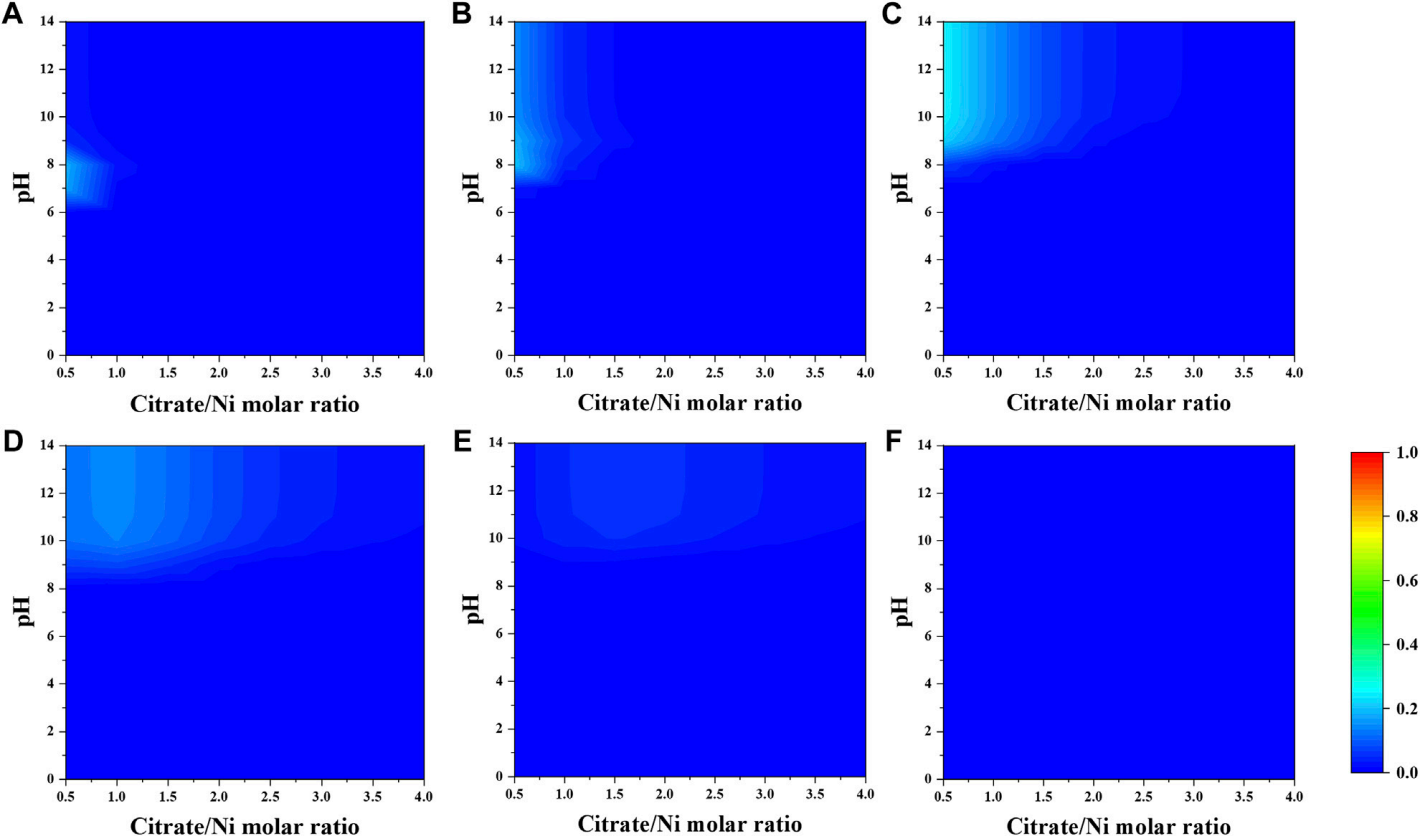
Fraction of Ni–ammonia complexes **(A)** [Ni(NH_3_)]^2+^, **(B)** [Ni(NH_3_) _2_] ^2+^, **(C)** [Ni(NH_3_) _3_] ^2+^, **(D)** [Ni(NH_3_) _4_] ^2+^, **(E)** [Ni(NH_3_) _5_] ^2+^, and **(F)** [Ni(NH_3_) _6_] ^2+^ as a function of citrate-to-nickel molar ratio and pH.

**FIGURE 4:**
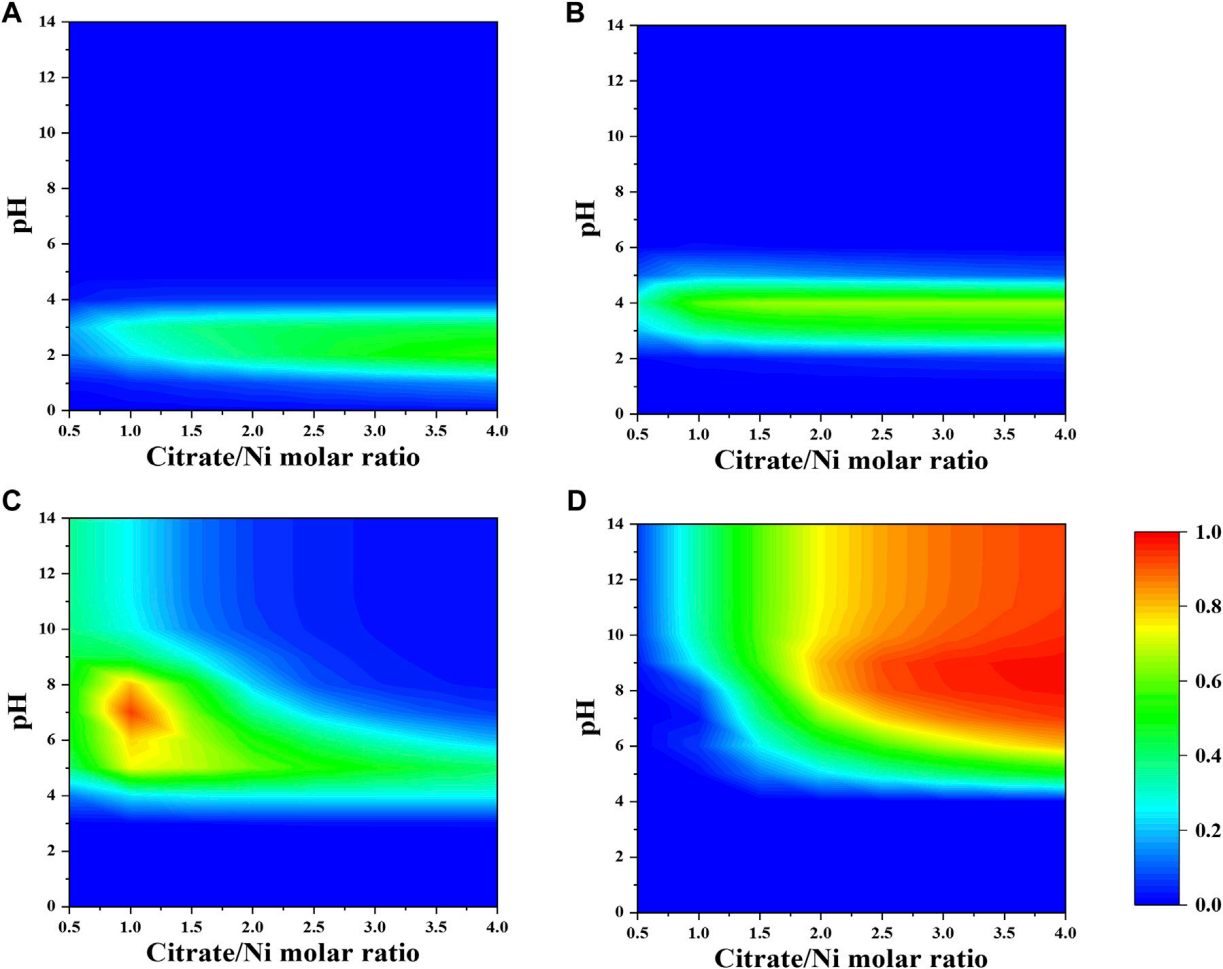
Fraction of nickel-citrate complexes **(A)** NiH_2_Cit^+^, **(B)** NiHCit, **(C)** NiCit^−^, and **(D)** NiCit_2_^4-^ as a function of citrate-to-nickel molar ratio and pH in the presence of NH_4_^+^.

When a fixed concentration of ammonium (e.g., 0.2 M) is introduced, citrate and ammonia are competing to complex nickel ions. In general, the fraction of nickel–ammonia complexes is relatively lower compared to other Ni–citrate complexes and optimized at lower range of nitrate-to-nickel ratio (Figures 3, 4). The largest fraction of nickel–ammonia complexes occurs at higher pH as the complexes contains larger number of ammonia molecules. Moreover, most nickel–ammonia complexes occur in the alkaline pH, which is also the favorable condition for NiCit^−^ and NiCit_2_^4−^species. Consequently, the incorporation of ammonium notably influences the fraction of NiCit^−^ and NiCit_2_^4-^, as suggested in Figure 4. Since ammonium does not have a significant effect on fraction of molybdenum species, the contour plots for molybdenum species in the presence of ammonium are not shown.

### Possible mechanisms of co-deposited Ni–Mo alloys

**FIGURE 5:**
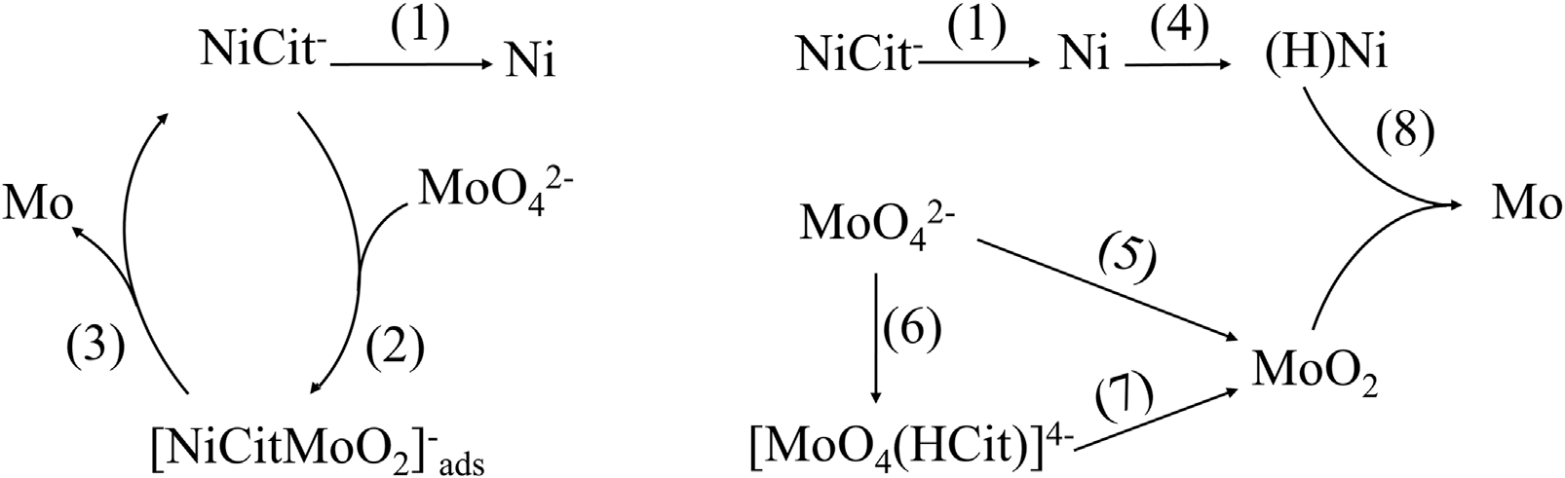
Two possible mechanisms of Ni–Mo co-deposition.

Provided the understanding about the distribution of complex species, it is crucial to determine the chemically active species for the reduction of Ni and Mo so that their fractions in the solution are maximized by controlling the precursor concentration and pH. Many different mechanisms of co-deposited Ni–Mo alloys were proposed; however, two mechanisms as shown in Figure 5 were commonly accepted by researchers for the co-deposition of Ni–Mo (Ernst et al., 1955; Ernst and Holt, 1958; Fukushima et al., 1979; Creager et al., 1982; Chassaing et al., 1989a; Crousier et al., 1992; Rengakuji et al., 1994; Podlaha and Landolt, 1997). For both reduction mechanisms, NiCit^−^ ion is the active species for the reduction of Ni as shown in the following equation (Safizadeh et al., 2015) (Podlaha and Landolt, 1997).

$$NiCit^{-}+2e\to Ni\left(s\right)+Cit^{-3}.$$ (4)

#### Mechanism 1

NiCit^−^ ion-dependent reduction of MoO_4_^2-^ (Podlaha and Landolt, 1997): the complete reduction of MoO_4_^2-^ ion into Mo comprises two constituent steps (Eqs 5, 6), in which NiCit^−^ ions act as a complexing agent. MoO_4_^2-^ ions were first partially reduced into MoO_2_ in the complexing form with NiCit^−^. Four electrons are required to form elemental Mo from MoO_4_^2-^.

$$\mathrm{Mo}O_{4}^{2-}+\mathrm{NiCi}t^{-}+2H_{2}O+2e^{-}\to {\left[\mathrm{NiCitMo}O_{2}\right]}_{\mathrm{ads}}^{-}+4OH^{-}$$ (5)

$${\left[\mathrm{NiCitMo}O_{2}\right]}_{\mathrm{ads}}^{-}+2H_{2}O+4e^{-}\to \mathrm{Mo}\left(s\right)+\mathrm{NiCi}t^{-}+4OH^{-}.$$ (6)

#### Mechanism 2

NiCit^−^ ion-independent reduction of MoO_4_^2-^ (Fukushima et al., 1979; Creager et al., 1982; Rengakuji et al., 1994): similarly, Mo (VI) in form of MoO_4_^2-^ is also reduced to Mo (IV) in MoO_2_ before becoming zero-valent Mo in the second possible mechanism. MoO_2_ can be generated from either MoO_4_^2-^ ion (Eq. 7) or complexing species [MoO_4_(HCit)]^4-^ (Eqs 8, 9). Being the intermediate of hydrogen gas evolution in equation (BeBtowska-Lehman, 1990), adsorbed hydrogen atoms on the deposited Ni surface are also the reducing agent to convert Mo (IV) to elemental Mo as shown in Eqs 11, 12.

• Partial reduction of Mo (VI) ions

$$\mathrm{Mo}O_{4}^{\mathrm{2-}}+4H_{2}O+2e^{-}\to \mathrm{Mo}O_{2}.2H_{2}O+4OH^{-},$$ (7)

$$\mathrm{Mo}O_{4}^{\mathrm{2-}}+\mathrm{HCi}t^{\mathrm{2-}}↔{\left[\mathrm{Mo}O_{4}\left(\mathrm{HCit}\right)\right]}^{4-},$$ (8)

$${\left[\mathrm{Mo}O_{4}\left(\mathrm{HCit}\right)\right]}^{4-}+H_{2}\mathrm{O+2e}\to \mathrm{Mo}O_{2}+\mathrm{HCi}t^{\mathrm{2-}}+3OH^{-}.$$ (9)

• Complete reduction to metallic Mo

$$2H_{2}\mathrm{O+2}e^{-}\to H_{2}\mathrm{+2O}H^{-},$$ (10)

$$H_{2}O+\mathrm{Ni}+e^{-}\to H\left(\mathrm{Ni}\right)\mathrm{+O}H^{-},$$ (11)

$$\mathrm{Mo}O_{2}\mathrm{+4H}\left(\mathrm{Ni}\right)\to \mathrm{Mo}\left(\mathrm{Ni}\right)\mathrm{+2}H_{2}O.$$ (12)

## Effect of ammonium/citrate complexing agent ratio

The deposition of Ni–Mo alloys is determined by not only the fraction of chemically active species but also the reduction at the interface of the substrate and electrolyte. Therefore, the electrolyte composition is selected to maximize the fraction of chemically active species, and Hull cell is used to study the effect of electrodeposition parameters on the formation of Ni–Mo alloys and Ni–Mo–O composites. The first systematically studied synthesis parameter is the complexing agents. The simulated fraction of species indicates that alkaline pH is appropriate to promote the chemically active nickel and molybdenum species. Experimental data from researchers also suggested the alkaline pH, commonly pH 9.5–11.5, for the deposition of metallic Ni–Mo alloys (Ernst et al., 1955; Ernst and Holt, 1958; Higashi et al., 1976; Arul Raj and Venkatesan, 1988; Nee et al., 1988; Chassaing et al., 1989a; Chassaing et al., 1989b). Therefore, to study the effect of ammonium-to-citrate molar ratio, the solution pH and concentration of nickel ions, molybdate ions, and citrate ions were fixed at pH 10, 0.1, and 0.05 M, respectively, while the concentration of ammonium ions was varied between 0 and 0.3 M.

**FIGURE 6:**
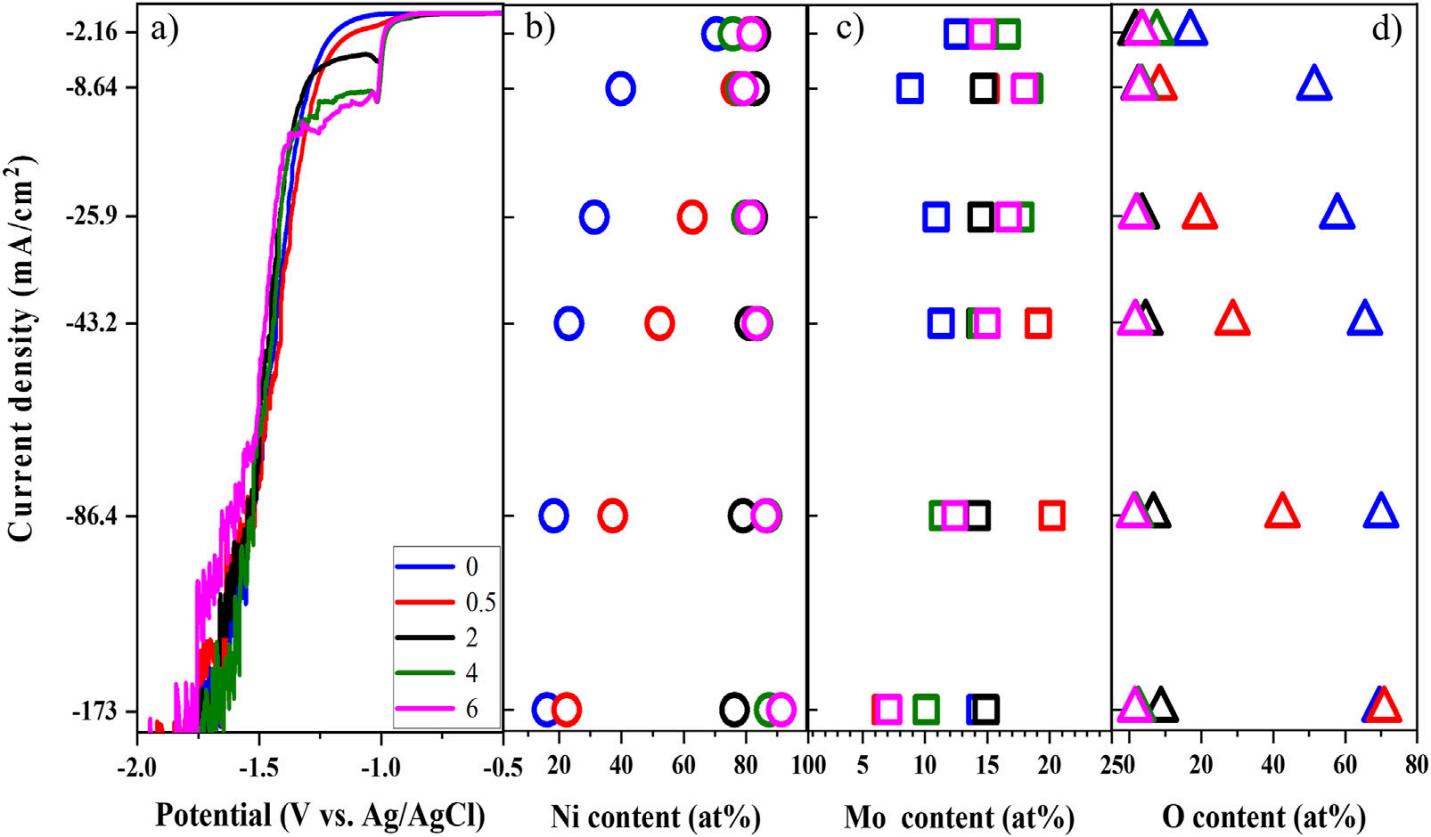
Linear sweep voltammetry **(A)**, Ni content **(B)**, Mo content **(C)**, and O content **(D)** of deposits as a function of ammonium-to-citrate molar ratio at fixed 0.1 M Ni^2+^, 0.05 M MoO_4_^2-^, and 0.05 M citrate and 25°C

The linear sweep voltammetry (LSV) in Figure 6A, which correlates the current density and applied potential, clearly shows the effect of complexing agent molar ratio. The shoulder and limited current become more pronounced and larger, respectively, as concentration of ammonium increases at the lower range of applied potential. The curves shift to more positive potential, implying the reduction of Ni–Mo requires less energy as the molar ratio increases. In other words, larger Ni–ammonia complex concentration might reduce the concentration overpotential, and more positive potential is thereby required for the same current density. The insignificant difference of polarization curves at more negative potential might be due to the dominance of HER over the reduction of Ni and Mo (Beltowska-Lehman et al., 2012). Utilizing galvanostatically deposited Ni–Mo alloys at 86.4 mA/cm^2^, the current efficiencies for complexing agent molar ratios of four and six are 28.0 and 31.9%, respectively, implying dominant side reaction and invariant effect of complexing agents at high-current density. Also, the two values are within the range of reported current efficiencies (BeBtowska-Lehman, 1990; Huang et al., 2015; Bigos et al., 2017). The details of measurement and calculation are discussed in the supplemental information*.*

The composition of deposits in Figures 6B–D suggests that the presence of ammonium is required for the formation of metallic Ni–Mo alloys or Ni–Mo–O composite with a low oxygen content. For the studied range of current density, the Ni content and O content generally increases and decreases, respectively, with higher ammonium concentrations. This suggests that the presence of ammonium ions promote the reduction of Ni. Moreover, a critical molar ratio of complexing agents above which the metallic Ni–Mo alloys were formed as shown in Figure 6D. On the other hand, the Mo content does not exhibit a monotonic change probably because Mo exists in the oxide form of different valent states. Ernst *et al.* reported that molybdate ions did not fully reduce to elemental molybdenum with six electrons in one step (Ernst and Holt, 1958). Instead, intermediates of molybdenum in the form of oxide or hydroxide were formed with different oxidation states. Moreover, no significant variation in the composition of deposits was observed at the ammonium concentrations of 0.2 and 0.3 M. XPS was carried out to examine the chemical oxidation states of Mo. The high-resolution core level binding energy of Mo 3 days from the ammonium/citrate molar ratio of 0–2 and 86.4 mA/cm^2^ are plotted in Supplementary Figure S3. Gaussian fitting was performed to deconvolute the peaks of Mo 3d_3/2_ and Mo 3d_5/2_. For the complexing agent molar ratio of 0 and 2, each of the Mo 3 days peaks splits into two peaks as illustrated in Supplementary Figures S3A,C. Elemental Mo is corresponding to the peaks at about 228 and 231 eV, while the 229 and 232 eV peaks are attributed to Mo^4+^ (Tian et al., 2015; Zhang et al., 2018). The deconvolution of Mo 3 days for the complexing molar ratio of 0.5 provides three peaks for each of Mo 3d_3/2_ and Mo 3d_5/2_. In addition to the peaks of Mo^0^ and Mo^4+^ from molar ratio of 0 and 2, the Mo^6+^ oxidation state is assigned to the two peaks Mo 3d_3/2_ (232.44 eV) and Mo 3d_5/2_ (235.59 eV) (Rao et al., 2016; Gao et al., 2017).

Ammonium ions mainly contribute to the complexation of nickel ions. Therefore, correlating the fraction of Ni complexes (i.e., Ni–citrate and Ni–ammonia) with the Ni content in deposits was conducted to elucidate the possible chemically active species for Ni reduction**.** Supplementary Figure S1A shows the changes of Ni–citrate complexes, in which the fraction of NiCit^−^ and NiCit_2_^4-^ have significant reduction and augmentation, respectively, with the increase in NH_4_^+^ concentration. On the other hand, the changes of Ni–ammonia complexes do not have a monotonic trend. Among them, the fraction of Ni(NH_3_)_3_^2+^ ion becomes larger with higher NH_4_^+^ concentration and roughly the same at 0.2 and 0.3 M NH_4_^+^. This is consistent with the changes of the Ni content observed in Figure 6B, indicating Ni(NH_3_)_3_^2+^ is probably the chemically active species for the reduction of Ni. This also explains the more negative limit current density at higher NH_4_^+^ concentration or more Ni(NH_3_)_3_^2+^ ions. As the dominant Mo species at pH 10, the concentration of MoO_4_^2-^ ions is fixed for different concentrations of NH_4_^+^. This might explain that the reduction of Mo is induced by the reduced Ni atoms. In other words, MoO_4_^2-^ ions can only be reduced partially if the reduced Ni atoms are not large enough. The partial reduction causes the presence of Mo in different oxidation states, which is shown in Figure 6C. Therefore, the reduction of the O content is probably attributed to the more complete reduction of Mo at larger concentration of ammonium. The role of the reduced Ni atoms is further confirmed by the formation of metallic Ni–Mo alloys at a high Ni content of deposit (Figures 6B,D). The experimental data suggests that the mechanism 2 is more practical, but the chemically active species for Ni reduction is probably Ni(NH_3_)_3_^2+^ rather than NiCit^−^. While the reduction of NiCit^−^ is under activation control (Cherkaoui et al., 1988; Chassaing et al., 1989a; Beltowska-Lehman et al., 2012), that of Ni(NH_3_)_3_^2+^ is probably under mass transport control.

The simulated fraction of Ni–ammonia complexes in Supplementary Figure S1B also suggests that abundance of ammonium ions leads to less chemically active Ni species and reduced Ni atoms. However, ammonium hydroxide has been commonly used to correct pH in many studies (Ernst et al., 1955; Ernst and Holt, 1958; Fukushima and Higashi, 1978; Chassaing et al., 1989a; Rengakuji et al., 1994; Chassaing et al., 2004; Donten et al., 2005; Beltowska-Lehman et al., 2012; Allahyarzadeh et al., 2016; Bigos et al., 2017). This might lead to the consideration of only Ni–citrate complexes as the chemically active species for Ni reduction. Ernst et al. (1955) claimed that excess ammonium ions resulted in a low Ni content of Ni–Mo alloy. A study of ammonium effect suggested that excess ammonium caused the reduction of the Mo content in the alloys (Podlaha and Landolt, 1996). Nevertheless, pH was not corrected, and depending on the ammonium concentration, meaning the fraction of complex species also varied. Conducting the effect of ammonium ion at lower pH of 8, Kuznetsov et al. (2005)found the formation of oxide occurred at the ammonium-to-citrate molar ratio above three. The smaller molar ratio might be attributed to smaller concentration of Ni–ammonia complexes at pH 8 in comparison to pH 10. The findings further support the concentration of NH_4_^+^ and Ni–ammonia complexes are crucial for the deposition of metallic Ni–Mo alloys.

**FIGURE 7:**
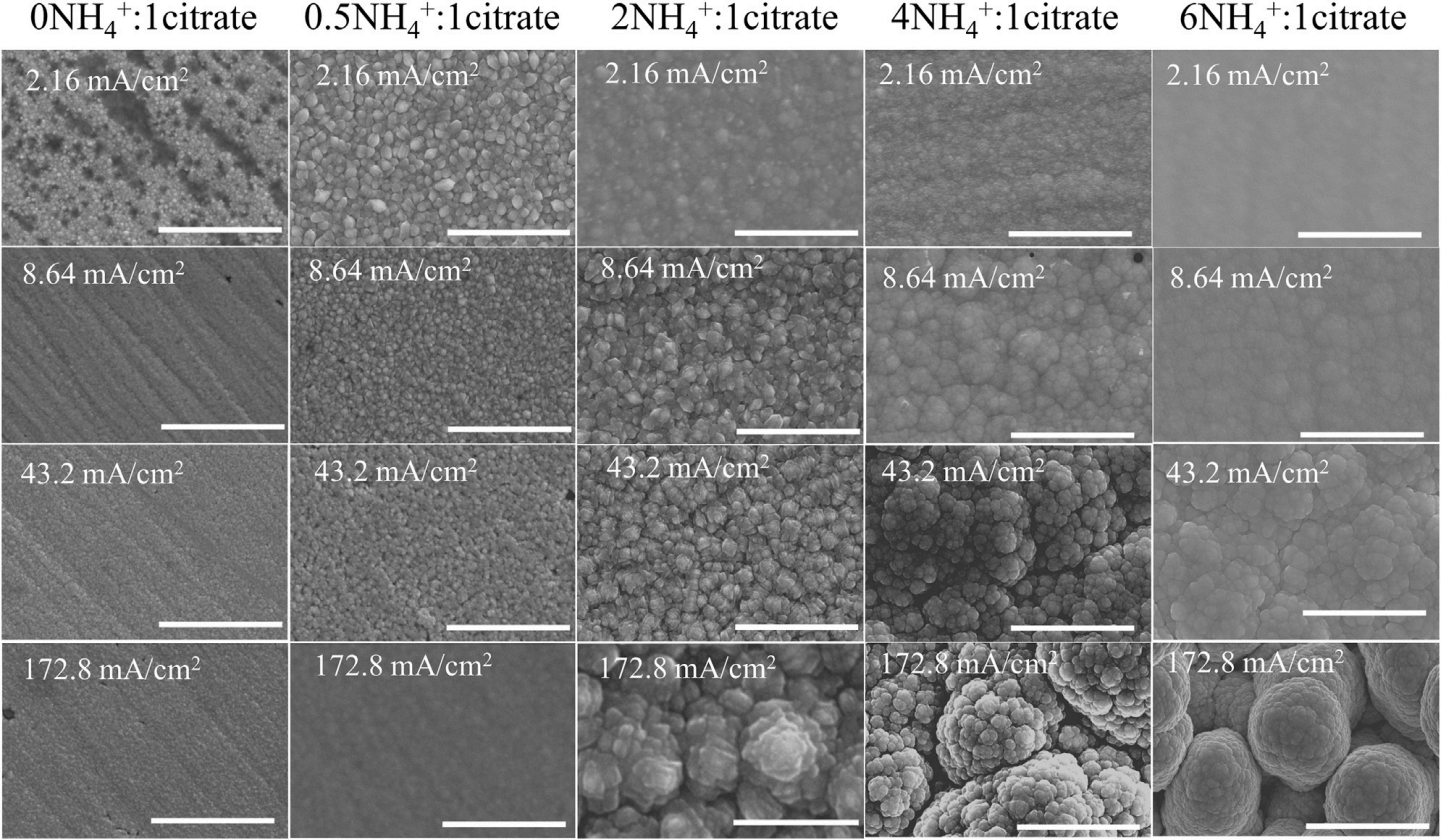
SEM images of deposits at different ammonium-to-citrate molar ratio and current density at fixed 0.1 M Ni^2+^, 0.05 M MoO_4_^2-^, 0.05 M citrate, and 0.2 M NH_4_^+^. Scale bar: 3 µm.

Morphology of the deposits was characterized by scanning electron microscopy (SEM). Four current densities of each condition were selected to show the alternation of morphology as shown in Figure 7. The structure of deposit generally transitioned from the smooth surface (amorphous oxides) to nodular structure (crystalline alloys) as the ammonium-to-citrate molar ratio increased. Different from the nodular structures of the two higher NH_4_^+^ concentrations, the dendritic structures at 0.1 M NH_4_^+^ seems to be the transition of the two trends of morphology evolution. The transition of morphology and preferential orientation of the films were further confirmed by the SEM cross-section images as shown in Supplementary Figure S2. The laminar growth corresponds to the smooth surface at the smaller molar ratio, while the nodular structure comes from the columnar growth at the higher ratio. The cross-sectional SEM images and EDS mapping of the deposits at 86.4 mA/cm^2^ in Supplementary Figure S2 indicate the molar ratio of two, which is the transition point for the growth mechanism. Donten et al. (2005) observed the transition of growth mechanism from the laminar to columnar growth as the Mo content of Ni–Mo alloys increased from the pyrophosphate bath. In addition, higher current densities resulted in the globular structure with larger roughness, which is independent of complexing agent molar ratio.

**FIGURE 8:**
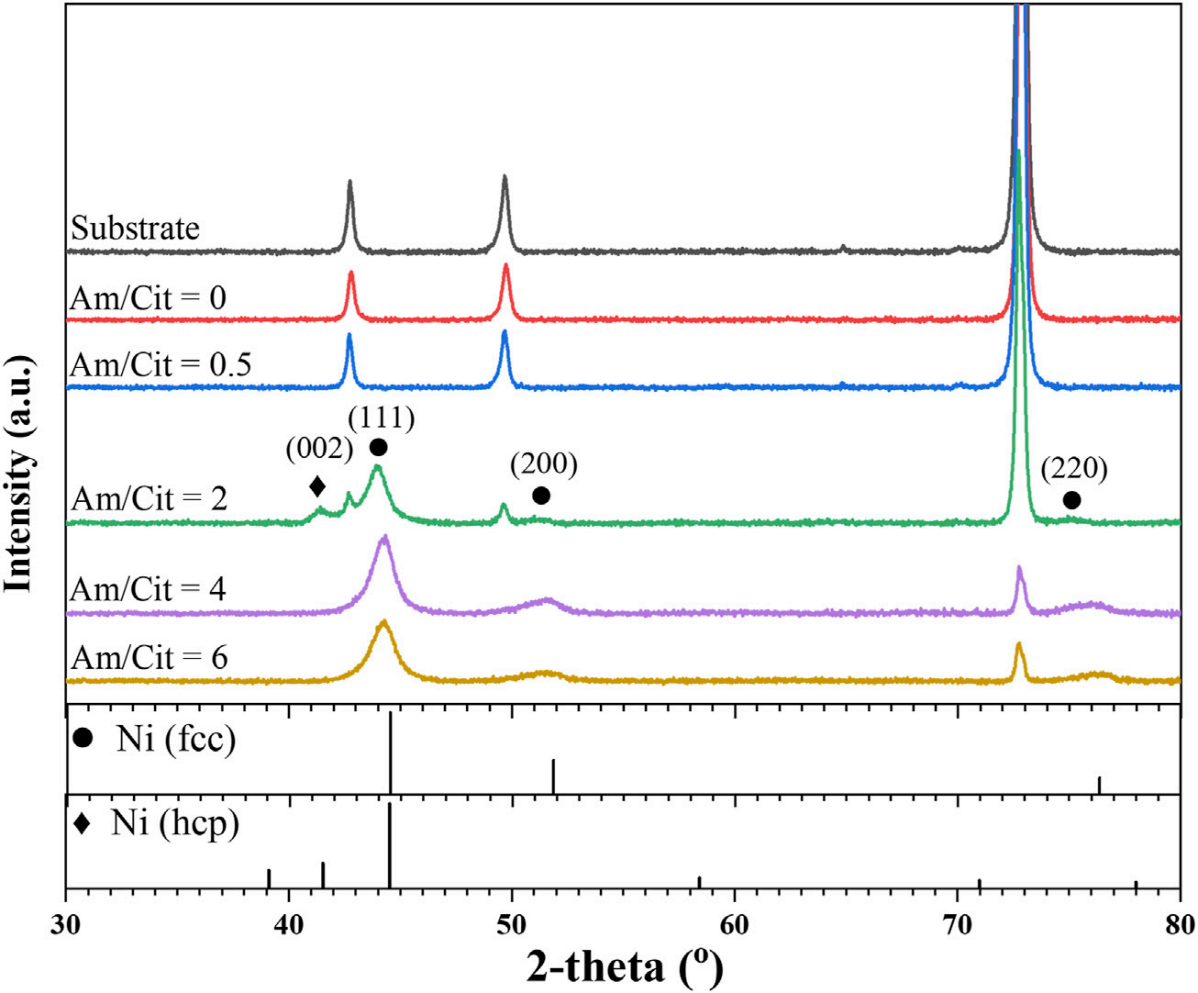
XRD spectra of deposits as a function of NH_4_^+^ concentration at fixed 0.1 M Ni^2+^, 0.05 M MoO_4_^2-^, 0.05 M citrate, and 0.2 M NH_4_^+^.

The samples at 86.4 mA/cm^2^ were also characterized by X-ray diffraction to correlate the crystal structure and the other material properties discussed earlier. As shown in Figure 8, the XRD patterns change with increase in the molar ratio of complexing agent, and the molar ratio of two is the transition point. At lower range of the molar ratio (i.e., 0–0.5), the peaks from the Ni–Mo–O deposit are not clearly exhibited, probably owing to the amorphous phase of the deposits. Bigos et al. (2017) suggested the low signal-to-noise ratio of X-ray reflections for nickel in the Ni–Mo alloys could be due to the small grains of atoms, which have the Ni typical orientation. In contrast, the XRD spectra indicate that the metallic Ni–Mo alloys have an fcc crystal structure for the higher range of molar ratio (i.e., 4–6). The two different molar ratios result in almost the same deposit composition (Figure 6) and XRD pattern (Figure 8), suggesting that deposit composition might be attributed to the crystal structure rather than the electrolyte composition. The 2-theta peaks of the samples were shifted to lower values in comparison to the JCPDS file#04–0850 for fcc-Ni phase. This might be owing to the insertion of Mo atoms into the lattice of Ni. As Mo atoms are larger than Ni atoms, the interplanar distances within the Ni lattice get expanded and lower the 2-theta values. Moreover, the broadening peaks suggest the deposits are in the nanocrystalline phase. Using the Scherrer equation, the calculated crystallite sizes were 19.1 nm for the hcp Ni and around 4.69–5.53 nm for the fcc Ni in the ammonium-to-citrate range of 2–6. Beltowska-Lehman et al. (2012) also reported the peak broadening and peaks shifting to lower 2-theta angle as the Mo content in the alloys became larger, corresponding to the formation of supersaturated solid solution in the nanocrystalline phase. As the transition point, the spectrum at the molar ratio of two not only appears the fcc-Ni phase but also the hcp-Ni phase (JCPDS file#45–1,027). The shift of 2-theta peaks to the left is also observed for this condition. The XRD data implies that amorphous and crystalline deposits have the smooth surface with laminar growth and the nodular structure with columnar growth, respectively.

## Conclusion

In conclusion, the fraction of species after complexation was simulated from pH and the concentration of metal precursors and complexing agents. While the fraction of Mo species is pH-dependent, the fraction of Ni species is determined by both pH and the initial concentration. The distribution of species predicts the solution composition and pH to maximize the fraction of chemically active species for Ni and Mo reduction. Provided the simulated fraction of species, the experimental data from studying the effect of ammonia-to-citrate molar ratio indicates that Ni(NH_3_)_3_^2+^ might be the chemically active species for Ni reduction under diffusion control. Excessive ammonium is not preferable for the formation of metallic alloys since it will reduce the fraction of Ni(NH_3_)_3_^2+^. The complexing molar ratio of two is a critical point for the transition from Ni–Mo–O to Ni–Mo alloys, corresponding the morphology changes from the smooth surface to globular structures. The growth mechanism converses from laminar to columnar growth. The Mo content and O content in the alloys and composites can be as large as 19 at% and 70 at%, respectively. At 86.4 mA/cm^2^, the alloys had current efficiency between 29.0 and 31.9%. Moreover, Mo in the composites had the oxidation state of 0 and 4, while the complexing agent molar ratio of 0.5 resulted in additional valence state of +6. The Ni–Mo–O deposits have the amorphous phase, while the metallic Ni–Mo alloys have the fcc phase. Furthermore, the induced co-deposition of Mo might be due to the reduced Ni and adsorbed H atoms.

## Data Availability

The original contributions presented in the study are included in the article/Supplementary Material; further inquiries can be directed to the corresponding author.
