# Supplementary material for: Effects of NH4 +/citrate complexing agent ratio on Ni–Mo and Ni–Mo–O electrodeposits from ammonium citrate baths
Source: Front Chem. 2022 Aug 30;10:942423. doi: 10.3389/fchem.2022.942423 (PMC9468647; doi:10.3389/fchem.2022.942423)
Supplement: Supplementary file 1 [file DataSheet1.docx]

**Supplementary Materials**

**Simulated fraction of complex species by MATLAB**

**Table S1.** Equilibrium constant of citrate and Mo (IV) ions

| **Complexation of citrate and Mo (VI) ions** |  |
| --- | --- |
| MoO_4_^2-^ + H^+^ + Cit^3-^ ↔ (MoO_4_)HCit^4-^ | 10^8.35^ |
| MoO_4_^2-^ + 2H^+^ + Cit^3-^ ↔ (MoO_4_)H_2_Cit^3-^ | 10^15^ |
| MoO_4_^2-^ + 3H^+^ + Cit^3-^ ↔ (MoO_4_)H_3_Cit^2-^ | 10^19.62^ |
| MoO_4_^2-^ + 4H^+^ + Cit^3-^ ↔ (MoO_4_)H_4_Cit^-^ | 10^21.12^ |
| 2MoO_4_^2-^ + 4H^+^ + Cit^3-^ ↔ (MoO_4_)_2_H_4_Cit^3-^ | 10^31.53^ |
| 2MoO_4_^2-^ + 4H^+^ + 2Cit^3-^ ↔ (MoO_4_)_2_H_4_Cit_2_^6-^ | 10^31.02^ |
| 2MoO_4_^2-^ + 5H^+^ + 2Cit^3-^ ↔ (MoO_4_)_2_H_5_Cit_2_^5-^ | 10^35.86^ |
| 2MoO_4_^2-^ +6 H^+^ + 2Cit^3-^ ↔ (MoO_4_)_2_H_6_Cit_2_^4-^ | 10^40.08^ |
| MoO_4_^2-^ + 4H^+^ + 2Cit^3-^ ↔ (MoO_4_)H_4_Cit_2_^4-^ | 10^25.34^ |
| MoO_4_^2-^ + 5H^+^ + 2Cit^3-^ ↔ (MoO_4_)H_5_Cit_2_^3-^ | 10^29.54^ |
| MoO_4_^2-^ + 6H^+^ +2 Cit^3-^ ↔ (MoO_4_)H_6_Cit_2_^2-^ | 10^33.34^ |
| 2MoO_4_^2-^ + 3H^+^ + Cit^3-^ ↔ (MoO_4_)_2_H_3_Cit^4-^ | 10^21.73^ |
| 2MoO_4_^2-^ + 4H^+^ + Cit^3-^ ↔ (MoO_4_)_2_H_4_Cit^3-^ | 10^26.9^ |
| 2MoO_4_^2-^ + 5H^+^ + Cit^3-^ ↔ (MoO_4_)_2_H_5_Cit^2-^ | 10^31.53^ |
| 4MoO_4_^2-^ + 9H^+^ + 2Cit^3-^ ↔ (MoO_4_)_4_H_9_Cit_2_^5-^ | 10^60.76^ |
| 4MoO_4_^2-^ + 10H^+^ + 2Cit^3-^ ↔ (MoO_4_)_4_H_10_Cit_2_^4-^ | 10^64.69^ |
| 4MoO_4_^2-^ + 11H^+^ + 4Cit^3-^ ↔ (MoO_4_)_4_H_11_Cit_4_^9-^ | 10^77.45^ |

**Algorithm derivation for MATLAB program**

The concentration of each species after complexation was obtained via the material balance of all metals and complexing agents in the electrolyte. For instance, a system of three non-linear equations for material balance was required for nickel, molybdenum, and citrate containing electrolyte. The equation started with setting the initial concentration of the precursors equal to the sum of concentration of all species present after complexation based on Tables 1-2 and S1. Concentrations of Ni^2+^, MoO_4_^2-^, and citrate ions at equilibrium and after complexation were considered as the common variables of the system. The concentrations of the complexes were substituted by these varibles and concentration of H^+^ (derivated from pH) via the equation of K constant for each complex. After the common variables were solved, the concentration as well as the fraction of all complexes were calculated and displayed as contour plots.

**Effect of ammonium/citrate complexing agent ratio**

**Figure S1**: Simulated fraction of Ni citrate complexes (a) and Ni ammonia complexes (b) as a function of ammonium-to-citrate molar ratio at fixed 0.1 M Ni^2+^, 0.05 M MoO_4_^2-^, 0.05 M citrate ions

**Cross-section SEM images and EDS analysis**


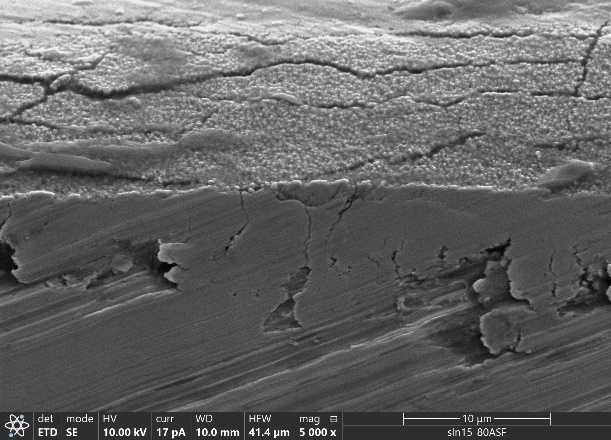

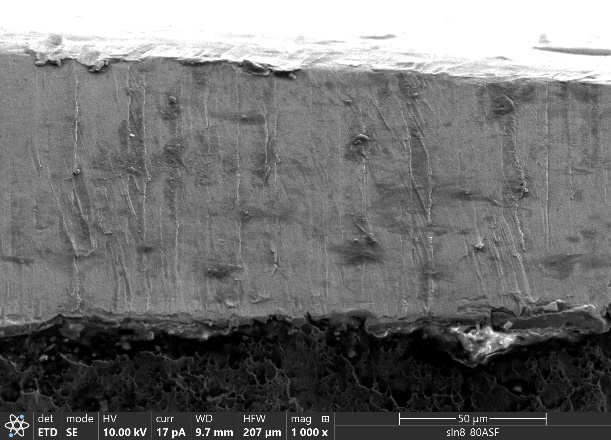

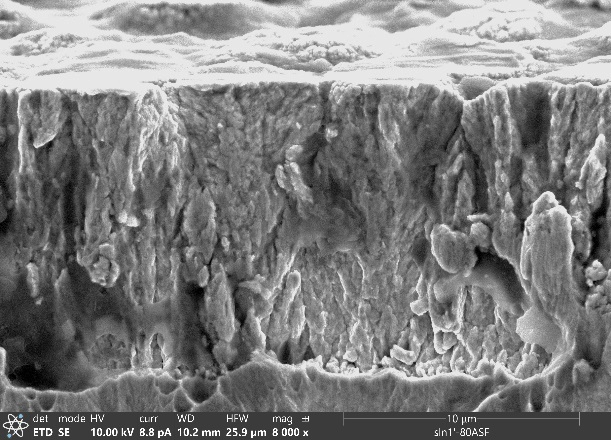

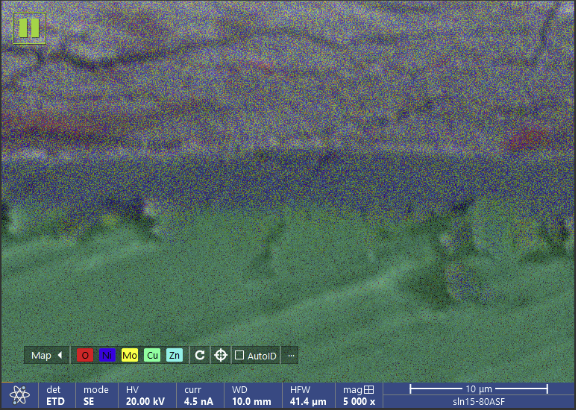


a)

d)

c)

b)

**Figure S2.** Cross-section SEM images of deposits at 86.4 mA/cm^2^ and different ammonium-to-citrate molar ratio a) 0, b) 2, c) 4 and d) EDS elemental mapping of SEM image b). Scale bar 10µm.

**Crystallite size of deposits calculated from XRD data**

| Ammonium-to-citrate ratio | Deposit phase | (hlk) | Crystallite size (nm) |
| --- | --- | --- | --- |
| 2 | HCP Ni | (002) | 19.1 |
| 2 | FCC Ni | (111) | 5.53 |
| 4 | FCC Ni | (111) | 5.21 |
| 6 | FCC Ni | (111) | 4.69 |

**Current efficiencies of metallic Ni-Mo alloy formation**

Typical three-electrode cell was employed for the galvanostatic deposition of metallic Ni-Mo alloys from the ammonium/citrate molar ratio of 4 and 6 at 86.4 mA/cm^2^. Nickel foil, platinum coated titanium plate and Ag/AgCl in 4M KCl served as the working, counter and reference electrode. After deposition, thickness of deposits was determined for the calculation of current efficiency as the following equations.

$$Current efficiency= \frac{Charge for Ni and Mo reduction}{Total charge}$$

$$Current efficiency= \frac{\#e transfer \times\# metal ions}{current density\times surface area\times reaction time}$$

$$\#metal ions= \frac{volume \times density of alloy}{molar mass of alloys}\times\% metal content\times Avogadro number$$

**X-ray photoelectron spectrometry**

**Figure S3**. XPS spectra for Mo 3d of deposits at 86.4 mA/cm^2^ and different ammonium-to-citrate molar ratio a) 0, b) 0.5, c) 2
